# The impact of chronic disease diagnoses on smoking behavior change and maintenance: Evidence from China

**DOI:** 10.18332/tid/176947

**Published:** 2024-01-23

**Authors:** Xinxin Chi, Xihua Liu, Cong Li, Wen Jiao

**Affiliations:** 1 Department of Economics, Qingdao University, Qingdao, China; 2 School of Business Administration, Zibo Vocational Institute, Zibo, China

**Keywords:** chronic disease diagnosis, prevention, smoking, heterogeneous treatment effects, LMICs

## Abstract

**INTRODUCTION:**

Managing chronic diseases and tobacco use is a formidable challenge in low- and middle-income countries (LMICs) with limited health literacy and access to quality healthcare. This study examines the empirical evidence from China, utilizing quasi-experimental approaches to assess the causal effect of chronic disease diagnoses on smoking behavior.

**METHODS:**

Employing the diagnosis of chronic disease in the older cohorts of the population as a natural experiment, this study utilizes recent advancements in difference-in-difference estimation methods (CS-DID) to investigate the effect of a diagnosis on smoking behavior. Self-reported new diagnoses of conditions ascertained chronic disease diagnoses. CS-DID was run using the study sample from the 2011 to 2018 waves of the China Health and Retirement Longitudinal Study, comparing results with traditional two-way fixed effects and event-study models.

**RESULTS:**

The average treatment effect (ATT) of CS-DID is slightly greater than the effects reported using conventional difference-in-difference methods. We found that diagnoses of cancer, heart disease, and stroke reduced smoking rates by 16% (95% CI: -24 – -8), smoking intensity by 0.31 (95% CI: -0.46 – -0.15), and had lasting impacts on smoking cessation behavior (one wave after diagnosis ATT= -0.17; 95% CI: -0.34 – -0.00, two waves after diagnosis ATT= -0.17; 95% CI: -0.37–0.03). A diagnosis of a mild chronic disease, such as hypertension, diabetes, asthma, chronic lung disease, liver disease, or gastric disease, had more negligible and transient effects on smoking behavior.

**CONCLUSIONS:**

Efforts to enhance smoking cessation in middle-aged and elderly patients with chronic diseases are crucial to improving health outcomes. The ‘teachable moment’ of chronic disease diagnosis should be seized to provide smoking cessation assistance to achieve the goal of healthy ageing.

## INTRODUCTION

The rapid change in population demographics toward ever-higher percentages of seniors poses unprecedented challenges to economic and social well-being worldwide. For low- and middle-income countries (LMICs) undergoing or have completed the epidemiological transition from infectious diseases to non-communicable diseases (NCDs), performing chronic disease control and prevention across the life course is essential for healthy ageing^1,2^. Smoking cessation is the most effective approach for reducing chronic disease morbidity, mortality, and medical burdens^3-5^. In recent years, researchers have begun to measure the effect of chronic disease diagnoses on smoking behavior based on population data. Most studies found that a diagnosis of chronic disease resulted in responses ranging from a reduction in the number of cigarettes smoked daily to complete smoking cessation in some but not all subjects^6,7^. In contrast, some studies found no effect of chronic disease on smoking behavior^8^; however, most of these studies employed data from high-income countries^9-12^. There are still very few studies conducted in low- and middle-income countries (LMICs), even though this is where 80% of smokers live^13^ and where chronic disease mortality rates are three times higher than in high-income nations^14^. In this study, we use Chinese data to supplement relevant studies in LMICs.

China is the world’s largest producer and consumer of tobacco products, accounting for about one-third of cigarettes in the world. There are more than 300 million smokers in China, of whom 1.4 million die from tobacco use every year. As many as half of Chinese adult males are smokers^15^. The illness, healthcare expenditure, and premature death caused by tobacco have inflicted substantial economic costs on the Chinese economy and negatively affect the wellbeing of the Chinese population^16^. In recent years, scholars have begun to explore the role of a diagnosis of a chronic disease on smoking behavior in China. Early cross-sectional evidence from rural residents in Shanghai suggests that smokers with tobacco-related chronic diseases have higher quit rates and quit attempt rates^17^. A study of panel data from the China Health and Nutrition Survey (2004–2015) showed that an increase in the number of chronic disease diagnoses among Chinese men aged ≥50 years led to slightly reduced smoking prevalence^18^. The above studies, based on morbidity, serve to illustrate the correlation between chronic disease and smoking behavior but cannot assess the causal effect^17^.

Some studies using data from high-income countries now adopt a quasi-experimental design to explore this causal relation, i.e. in comparisons of smoking behavior in samples with and without a diagnosis of chronic disease^12,19^. Similar studies of causal inference using Chinese data are rare. To the best of our knowledge, only two comparable publications are available^20,21^; both studies defined a new chronic disease diagnosis as a health shock variable between pre- and post-diagnosis surveys, to minimize reverse causality. One of the earlier studies used a random effects model to find that health shocks (high blood pressure, diabetes, myocardial infarction, lung cancer, asthma, and stroke or transient ischemic attack) reduced smoking prevalence by 10% in the short-term in China from 1991 to 2011, with long-term effects comparable to the short-term effects^20^. The more recent article applied a multilevel propensity score match difference-in-difference to analyze the China Health and Retirement Longitudinal Study (CHARLS) data in 2015 and 2018. The results showed that after major health shocks (cancer, heart disease, stroke), smoking rates were 0.59 times less in the shocked group than in the non-shocked group, and for minor health shocks (hypertension, diabetes), the ratio was 0.74^21^.

In the present study, we used longer term CHARLS data (2011–2018) and examined a broader range of disease types. Furthermore, recent developments in econometrics suggest that traditional DID methods cannot handle the staggered timing of chronic disease diagnoses^22^. Commonly used two-way fixed effects (TWFE) and event-study methods can cope with endogeneity but cannot address treatment heterogeneity. A modified difference-in-difference proposed by Callaway and Sant’Anna^23^ (referred to as CS-DID) can solve treatment heterogeneity caused by the staggered occurrence of chronic disease diagnoses. As such, we use the traditional TWFE and event-study models along with the improved CS-DID approach and compare results.

## METHODS

### Data and sample

This study used data from the 2011–2018 China Health and Retirement Longitudinal Study (CHARLS)^24^. CHARLS is a longitudinal survey that covered 28 provinces with substantial variations in personal information, household structure, economic support, health status, medical service utilization, medical insurance, and work situation in 2011, 2013, 2015, and 2018. CHARLS covered 23000 respondents in 12400 households in 2015, which generally represents the middle-aged and elderly population in China, and includes common chronic diseases and detailed smoking behavior data, which provides excellent data support for this study.

**Figure 1:**
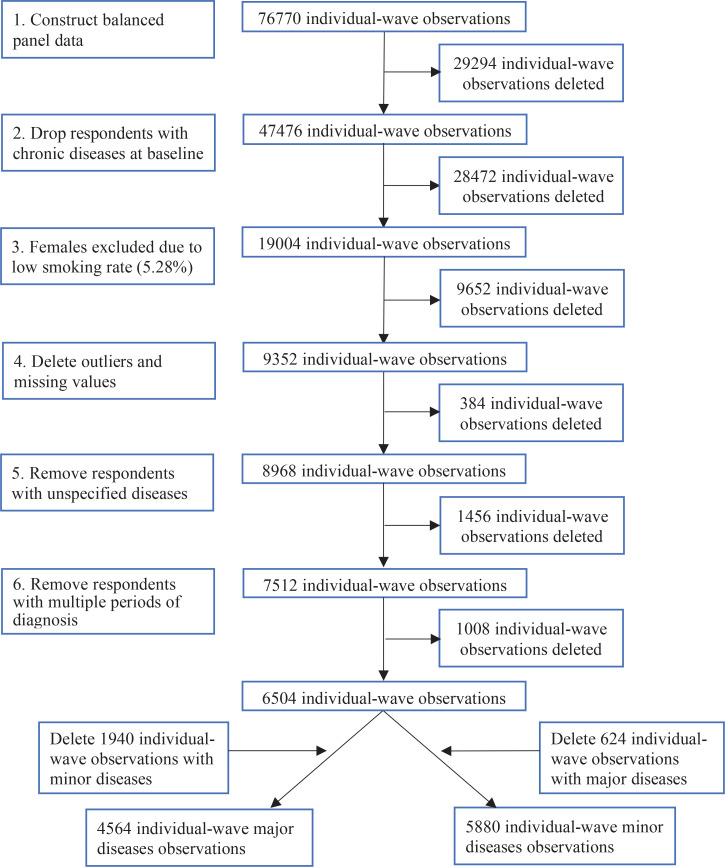
Flow chart of data processing

For the study’s objectives, we used a variety of sample limitations and data cleaning techniques to create our analysis sample, including: 1) the construction of balanced panel data which retained only individuals surveyed in all four survey periods, to more accurately assess the dynamic effects of chronic disease diagnoses; 2) respondents with chronic diseases at baseline were dropped to exclude pre-study interference; 3) we excluded female samples due to the extremely low percentage (5.28%) of female smokers in the data; 4) respondents aged <45 or >80 years and responses missing values for the number of cigarettes per day and covariates were deleted; 5) as CHARLS queries a variety of chronic diseases, we selected nine chronic diseases related to smoking and removed respondents who reported unrelated or unspecified chronic diseases; 6) multiple diagnoses over waves can affect the assessment of dynamic effects, so we removed respondents with multiple periods of chronic disease diagnosis. These preliminary techniques yielded a balanced panel data of 6504 individual-wave observations for 1626 respondents; and 7) to study the effect of major chronic diseases, we removed 1940 observations with minor chronic diseases to net out the effect of minor conditions. Similarly, we excluded 624 observations diagnosed with major chronic diseases when studying the effect of minor chronic diseases. A flow chart of data processing is shown in Figure 1.

### Variables

The primary outcome of interest was whether the respondent smoked or not, coded as 0 if the respondent reported he did not smoke in the current reporting period and 1 if he still smoked. We further examined daily cigarette consumption to assess whether a diagnosis of chronic disease impacted the degree of tobacco addiction. The variable was recorded as an ordinal variable to measure smoking intensity: 0=no smoking; 1=light smoking (1–10 cigarettes); 2=moderate smoking (11–20 cigarettes); and 3=heavy smoking (≥21 cigarettes).

The independent variable was a dichotomous indicator of whether the respondent had a chronic disease diagnosis. Because chronic diseases tend to be of long duration and are difficult to cure, we assume that individuals would continue to suffer from their chronic diseases after their initial diagnosis. The independent variable was assigned the value of 0 before the participant reporting a diagnosis of chronic disease and 1 during the period of reporting the diagnosis and thereafter. The chronic diseases examined include hypertension, diabetes, cancer, heart disease, stroke, asthma, chronic lung disease, liver disease, and gastric diseases, incidences of which are all correlated to smoking^17^. Referring to previous studies^21,25^, we divided chronic diseases into major and minor categories to check the heterogeneity of disease severity. Respondents were coded as having a major chronic episode if they reported new cancer, heart disease, or stroke, usually severe and acute. Also, if respondents reported new hypertension, diabetes, asthma, chronic lung disease, liver disease, and gastric disease, a category of mild and manageable short-term conditions, these were recorded as minor episodes.

We selected covariates based on Andersen’s behavioral model. This model incorporates multiple factors that may influence health and illness behavior into a concise analytical framework, concluding that predisposing factors (age, etc.), enabling resources (financial situation, etc.), and demand factors (illness, etc.) jointly contribute to one’s medical and preventive behaviors^26^. For predisposing factors, this study controlled for age, marital status, education level, and work status. Regarding enabling factors, we chose: the residential area (i.e. urban, rural), household expenditure per capita (PCHE), and medical insurance situation. As for need factors, mental status, activities of daily living (ADL), and self-rated health (SRH) are selected. Mental status is represented by the Center for Epidemiological Studies Depression Scale (CES-D) score, with values ranging 0–30, with higher values indicating more severe depressive symptoms. Individuals are considered to have impairment in ADL if they have difficulty with any of the twelve activities: toileting, eating, dressing, controlling bowel movements, getting up and out of bed, bathing, shopping, talking on the phone, cooking, housework, taking medication, and managing finances.

### Statistical analysis

Descriptive statistics illustrate changes in smoking status, chronic disease prevalence, and covariates across four CHARLS waves, where qualitative data are expressed as frequency (n) and proportion (%), and quantitative data are presented as mean and standard deviation (SD). Our overall causal effect analysis method was DID. We compared differences in changes in smoking behaviors before and after diagnosis between people with and without chronic disease. The DID analysis is essentially a quasi-experimental approach that uses a control group (i.e. non-diagnosed individuals’ smoking behavior changes) to act as a counterfactual for what would have been anticipated to happen without a specific treatment (i.e. the diagnosis)^27^. We used the TWFE model to identify causal effects in the baseline model. The fundamental assumption of the DID approach is that smoking trends remained consistent between the groups with and without chronic diseases in the period before the diagnosis. We then used the event-study approach to test the parallel trends assumption. Event-study regression is similar to the interrupted time series analysis; it takes the period just before the event as a counterfactual comparison group and enables assessment of the dynamic effects of chronic disease diagnoses.

The sample sizes of the treatment and control groups differed across periods (Supplementary file Table 1), which reflects that individuals are not diagnosed with chronic conditions at the same time. There was group and temporal heterogeneity in treatment effects. In the TWFE framework, the core estimated coefficients are a weighted average of multiple sets of underlying two-group, two-period difference-in-difference (2×2 DID) estimates (see the Supplementary file for specific sets)^22^. The estimated effect of DID in the post-disease sample, based on the pre-disease sample as the control group, would be underestimated because the effect of a chronic disease diagnosis on smoking behavior does not disappear immediately^23^. To overcome this issue, we further use the CS-DID to estimate the TWFE model with multiple periods. The CS-DID framework averages all 2×2 DID treatment effects based on the weighting scale of the group size. This simple-aggregated effect can rule out the potential negative weight issues of the TWFE model. The dynamic effects in the framework are averages of all 2×2 DID treatment effects at different lengths of treatment, which provide better alternatives for event study.

All analyses were conducted in STATA 17.0 (Stata Corp, College Station, TX) at a two-tailed 5% significance level. For comparison, we estimated all models using ordinary least squares regression with fixed effects for individual and year. We clustered standard errors by the individual to account for inter-individual correlation over time. We used logarithms of age, PCHE, and CES-D scores, to mitigate potential negative impacts of larger orders of magnitude.

## RESULTS

Table 1 gives the descriptive statistics of smoking status, chronic disease prevalence, and covariates for each survey wave. The table shows that the smoking rate of the survey sample is around 60%, and the average number of cigarettes smoked per day is about 18, showing a decreasing trend. The prevalence of chronic diseases increases with age from period to period. The average age of the initial surveyed sample was 57 years, generally with health insurance. Additionally, the proportion of respondents who are unemployed or have impairment in daily living activities increases yearly.

**Table 1:** Descriptive statistics of smoking status, chronic disease prevalence, and covariates across four CHARLS waves (2011, 2013, 2015, 2018) (N=1626)

*Characteristics*	*2011*	*2013*	*2015*	*2018*
	*n (%)*	*n (%)*	*n (%)*	*n (%)*
**Smoking status**				
Yes	1036 (0.637)	1031 (0.634)	962 (0.592)	921 (0.566)
No	590 (0.363)	595 (0.366)	664 (0.408)	705 (0.434)
Daily cigarettes, mean (SD)	18.982 (12.013)	18.809 (12.221)	18.634 (12.260)	18.262 (11.934)
**Major chronic disease**				
Yes		18 (0.011)	19 (0.012)	119 (0.073)
No		1608 (0.989)	1607 (0.988)	1507 (0.927)
**Minor chronic disease**				
Yes		79 (0.049)	106 (0.065)	300 (0.185)
No		1547 (0.951)	1520 (0.935)	1326 (0.815)
**Age** (years), mean (SD)	57.322 (8.245)	59.322 (8.245)	61.322 (8.245)	64.322 (8.245)
**Married**				
Yes	1514 (0.931)	1505 (0.926)	1485 (0.913)	1450 (0.892)
No	112 (0.069)	121 (0.074)	141 (0.087)	176 (0.108)
**Having a job**				
Yes	1394 (0.857)	1363 (0.838)	1286 (0.791)	1219 (0.750)
No	232 (0.143)	263 (0.162)	340 (0.209)	407 (0.250)
**Residence**				
Rural	1089 (0.670)	1089 (0.670)	1347 (0.828)	1315 (0.809)
Urban	537 (0.330)	537 (0.330)	279 (0.172)	311 (0.191)
**Household expenditure per capita**, mean (SD)	6488.264 (5480.378)	9274.384 (7175.662)	11649.685 (10987.662)	13086.898 (12891.853)
**Having medical insurance**				
Yes	1523 (0.937)	1565 (0.962)	1582 (0.973)	1576 (0.969)
No	103 (0.063)	61 (0.038)	44 (0.027)	50 (0.031)
**CES-D scores**, mean (SD)	8.066 (4.193)	6.606 (4.294)	7.213 (4.476)	6.430 (5.372)
**Having activities of daily life (ADL) impairment**				
Yes	211 (0.130)	263 (0.162)	271 (0.167)	320 (0.197)
No	1415 (0.870)	1363 (0.838)	1355 (0.833)	1306 (0.803)
**Self-rated health**				
Good	1457 (0.896)	1503 (0.924)	1440 (0.886)	1410 (0.867)
Poor	169 (0.104)	123 (0.076)	186 (0.114)	216 (0.133)

### Results of the TWFE analyses

Table 2 displays the effect of chronic disease diagnoses on smoking behavior from the TWFE models. For major chronic conditions, smoking rates decreased by 13% (95% CI: -19 – -6), and smoking intensity fell by 0.28 (95% CI: -0.42 – -0.14). The effect of minor chronic diseases was negative but not statistically significant (smoking, average treatment effect ATT= -0.02; 95% CI: -0.05–0.01, smoking intensity ATT= -0.03; 95% CI: -0.10–0.03).

**Table 2:** Estimated effects of chronic disease diagnoses on smoking and smoking intensity among Chinese middle-aged and elderly people based on TWFE analyses^†^ (major chronic diseases, N=4564; minor chronic diseases, N=5880)

	*Major chronic diseases ^a^*		*Minor chronic diseases ^b^*	
	*Smoking*	*Smoking intensity*	*Smoking*	*Smoking intensity*
	*ATT (95% CI)*	*ATT (95% CI)*	*ATT (95% CI)*	*ATT (95% CI)*
Estimated causal effect	-0.13*** (-0.19 – -0.06)	-0.28*** (-0.42 – -0.14)	-0.02 (-0.05–0.01)	-0.03 (-0.10–0.03)
Log (age)	1.07 (-0.47–2.61)	3.47** (0.33–6.60)	1.10 (-0.24–2.44)	3.50*** (0.86–6.14)
Married	-0.03 (-0.08–0.03)	-0.17** (-0.30 – -0.04)	-0.03 (-0.08–0.02)	-0.14** (-0.26 – -0.01)
Job	0.01 (-0.02–0.04)	0.03 (-0.04–0.09)	0.01 (-0.01–0.04)	0.04 (-0.02–0.10)
Residence	-0.00 (-0.04–0.03)	-0.02 (-0.09–0.05)	-0.01 (-0.04–0.02)	-0.01 (-0.07–0.05)
Log (PCHE)	0.01 (-0.01–0.02)	0.02 (-0.01–0.05)	0.00 (-0.01–0.01)	0.01 (-0.01–0.03)
Medical insurance	-0.03 (-0.07–0.01)	-0.00 (-0.11–0.10)	-0.03 (-0.07–0.01)	-0.03 (-0.13–0.06)
Log (CES-D score)	-0.00 (-0.01–0.01)	0.01 (-0.01–0.04)	0.00 (-0.01–0.01)	0.02* (-0.00–0.05)
ADL	-0.01 (-0.04–0.01)	-0.03 (-0.09–0.03)	-0.01 (-0.03–0.02)	-0.02 (-0.07–0.03)
SRH	-0.03 (-0.07–0.01)	-0.10*** (-0.17 – -0.02)	-0.03** (-0.06 – -0.00)	-0.07** (-0.14 – -0.01)
Individual fixed effects	Yes	Yes	Yes	Yes
Time fixed effects	Yes	Yes	Yes	Yes
Adjusted R^2^	0.78	0.79	0.77	0.79

a Sample with minor chronic diseases is excluded from the estimates.

b Sample with major chronic diseases is excluded from the estimates.

† TWFE: two-way fixed effects.

ATT: average treatment effect. PCHE: per capita household expenditure.

* p<0.1,

** p< 0.05,

*** p<0.01.

### Results of the event-study analyses

**Figure 2:**
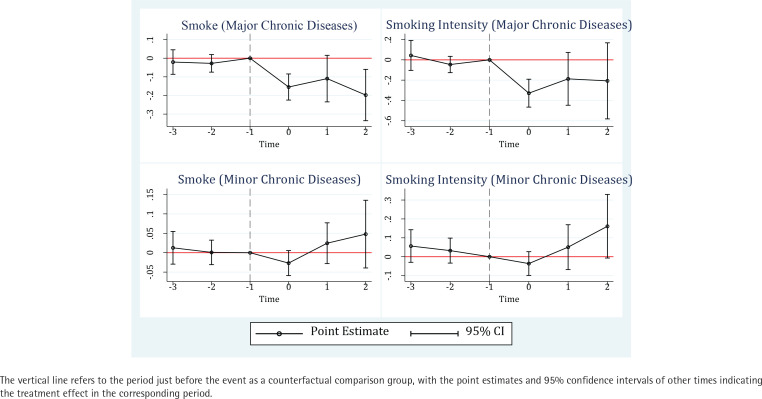
Event-study estimates for chronic disease diagnosis on smoking and smoking intensity among Chinese middle-aged and elderly people (for major chronic diseases, N=4564; for minor chronic diseases, N=5880)

We further investigate the dynamic effect of a diagnosis of chronic disease on smoking behavior using the event-study method, and the coefficients are depicted in Figure 2. The pre-diagnosis coefficients are not statistically significant. For smoking and cigarette counts, the corresponding p-values of an F-test for the common assumption that all pre-diagnosis coefficients are zero are 0.46 and 0.33 for major illness, 0.83 and 0.40 for minor illness. All coefficients for the pre-diagnosis were statistically insignificant, also validating the parallel trend assumption. Moreover, the significant negative coefficients for the post-diagnosis in the upper left plot reveal persistent changes in smoking behavior after cancer, heart disease, and stroke in the current and second periods of diagnosis. In contrast, the effect of minor chronic diseases on reducing smoking is not significant, and the daily cigarette consumption even increased significantly in the two periods after diagnosis.

### Results of the CS-DID analyses

Given the time-varying occurrence of chronic disease diagnosis, we employed the CS-DID method for further scrutiny. The simple-aggregated effect is equivalent to the ATT in the TWFE model, and it represents the average of all group-time treatment effects, with weights proportional to group size. The event time in dynamic effects shows the relative time to chronic disease diagnosis. For example, -1 shows one wave before diagnosis, and 0 shows the wave of diagnosis. The dynamic effects indicate no change in smoking behavior prior to diagnosis. A diagnosis of major diseases motivates patients to quit smoking (ATT= -0.16, 95% CI: -0.24 – -0.08), and the effect is valid in the long-term (one wave after diagnosis ATT= -0.17; 95% CI: -0.34 – -0.00, two waves after diagnosis ATT= -0.17; 95% CI: -0.37– 0.03). Note that while the treatment effect for the two post-diagnosis periods is not significant at the 5% level, it is significantly negative at the 10% level, suggesting a long-term trend in the effect of major chronic disease diagnosis on smoking cessation behavior. By comparison, the impact of smoking intensity is only significant in the short-term (for the current period of diagnosis ATT= -0.34; 95% CI: -0.49 – -0.20, one wave after diagnosis ATT = -0.24; 95% CI: -0.57–0.10, two waves after diagnosis ATT= -0.15; 95% CI: -0.58–0.28). The treatment effect for minor disease diagnosis is also insignificant (smoking ATT= -0.00; 95% CI: -0.04–0.04, smoking intensity ATT=0.02; 95% CI: -0.06–0.09), and dynamic effects show that the smoking intensity among individuals with minor chronic disease is higher (ATT=0.24; 95% CI: 0.04–0.45) after being treated for two periods than in the non-illness group (Table 3).

**Table 3:** Estimated effects of chronic disease diagnoses on smoking and smoking intensity among Chinese middle-aged and elderly people based on CS-DID analyses^†^ (major chronic diseases, N=4564; minor chronic diseases, N=5880)

*Effects*	*Major chronic diseases ^a^*		*Minor chronic diseases ^b^*	
	*Smoking*	*Smoking intensity*	*Smoking*	*Smoking intensity*
	*ATT (95% CI)*	*ATT (95% CI)*	*ATT (95% CI)*	*ATT (95% CI)*
**Simple-aggregated effect**	-0.16*** (-0.24 – -0.08)	-0.31*** (-0.46 – -0.15)	-0.00 (-0.04–0.04)	0.02 (-0.06–0.09)
**Dynamic effects**				
**Event time**				
-2	0.01 (-0.05–0.07)	-0.07 (-0.21–0.08)	-0.01 (-0.05–0.03)	-0.03 (-0.12–0.05)
-1	0.03 (-0.01–0.08)	0.07 (-0.02–0.15)	0.01 (-0.03–0.04)	-0.01 (-0.08–0.05)
0	-0.16*** (-0.23 – -0.09)	-0.34*** (-0.49 – -0.20)	-0.03* (-0.06–0.00)	-0.05 (-0.11–0.02)
1	-0.17** (-0.34 – -0.00)	-0.24 (-0.57–0.10)	0.04 (-0.02–0.09)	0.09 (-0.05–0.22)
2	-0.17* (-0.37–0.03)	-0.15 (-0.58–0.28)	0.09 (-0.02–0.19)	0.24** (0.04–0.45)

a Sample with minor chronic diseases is excluded from the estimates.

b Sample with major chronic diseases is excluded from the estimates.

ATT: average treatment effect.

† CS-DID: A modified difference-in-difference^23^.

* p<0.1,

** p< 0.05,

*** p<0.01.

## DISCUSSION

Our study rigorously assessed the impact of chronic disease diagnosis on smoking behavior change and maintenance. Differing from previous studies, we noted group and temporal heterogeneity in treatment effects and introduced a modified DID approach (CS-DID) proposed by Callaway and Sant’Anna^23^ to obtain ‘cleaner’ treatment effects. Consistent with the results of the conventional TWFE and event-study, the CS-DID estimates suggested that major chronic disease diagnoses reduce smoking probability and quantity, and minor chronic diseases have a weaker effect on smoking reduction and cessation. In particular, measurable changes in behavior after major diagnoses estimated by CS-DID exceeded those of conventional quasi-experimental methods due to the resolution of underestimation problems arising from group and temporal heterogeneity.

We estimated a 16% short-term reduction in smoking prevalence for individuals with major chronic diseases, a measure that falls between two similar studies in China^20,21^. For minor chronic diseases, the average treatment effect of 3% was much smaller than that found in the previous study^21^. This may be accounted for by voluntary changes in the behavior of middle-aged and elderly Chinese who have become more aware of the dangers of smoking, compared to the past. Therefore, the more recent the data used, the larger the estimated effect. In addition, the disease types used in these studies also varied. Compared with previous studies, we assessed the long-term effects of a chronic disease diagnosis using a newer and more refined approach. We found a measurable reduction in smoking prevalence after a diagnosis of a major chronic disease but a non-sustained effect on smoking intensity. Decreases in intensity were a prelude to smoking cessation, and some patients reduced their smoking intensity in the current period of diagnosis but did not achieve complete cessation. The significantly positive coefficient for smoking intensity in the two post-minor-diagnosis periods may be because the treated group was more addicted to cigarettes and relapsed into their smoking habit after they experienced an improvement in their condition. However, due to the short panel, the insights into trends in the impact of diagnosis are limited. For example, the effect of diagnosis after two periods was obtained based on only the cohort reporting a new diagnosis in the 2013 survey. Therefore, these data do not provide robust insights into the overall impact of a diagnosis after about five years since the diagnosis was received.

In high- and middle-income countries, the quit rate of smokers usually exceeds 50%^28^, while in China, the figure is only 10%^29^, and Chinese smokers report a low intention to quit and demonstrate high nicotine addiction, which implies a high probability of relapse after quitting^30^. The overall smoking cessation rate due to major chronic diseases estimated in this study was 16%, which was slightly higher than average but insignificant in relation to combating the growing prevalence of chronic diseases. Avoiding tobacco is essential for the secondary prevention of non-communicable chronic diseases, as reflected in the World Code Against Cancer Framework and the Global Hearts Hypertension Control Program, both of which include avoiding tobacco as the first code for disease prevention^31,32^.

A diagnosis of chronic disease can be used as a ‘teachable moment’ for providing tobacco control education and health guidance to smokers. In China, complete smoking cessation as a response to a diagnosis of chronic disease occurs only in a limited number of patients, indicating that the role of smoking cessation in secondary and tertiary prevention of chronic diseases has not been emphasized. Some individuals decreased their smoking intensity only in the short-term, indicating that the ‘O’ measure of MPOWER was not effectively implemented in China.

The most recent survey based on Chinese data suggests that smoking increases the risk of developing 56 diseases and dying from 22 diseases, including many diseases not empirically thought to be related to smoking, such as peptic ulcers, cataracts, and metabolic disorders^33^. Prolonged smoking cessation is necessary to reduce chronic disease morbidity and mortality^34,35^. However, our study suggests that middle-aged and older adults in China lack awareness of chronic disease control and prevention, possess limited knowledge of the dangers of tobacco, and have insufficient motivation to quit. This is a problem common to many LMICs in epidemiological transition. Further implementation of MPOWER and multilevel prevention of chronic diseases across the life course in these countries is urgent.

### Strengths and limitations

The study used a quasi-experimental design to assess the change and maintenance of smoking behavior after chronic disease diagnosis. Methodologically, multiple and most advanced methods were used to obtain ‘cleaner’ causal treatment effects than older studies. Specifically, we adopted the most advanced CS-DID model that considers endogeneity and the heterogeneous treatment effects from staggered time. Content-wise, our study illustrated the heterogeneity of the impact of major and minor conditions and analyzed the maintenance of behavioral changes. This study contributes to the literature examining the causal effect of chronic disease diagnosis on smoking behavior in LMICs.

There are several potential limitations of this study. First, this study was based on data from an interval of two years or more. The smoking behavior of the sample may have changed multiple times during the two years, and we cannot capture these changes. Second, we had not studied the effect of a specific single disease on smoking due to the small sample with a single chronic disease. Third, the data included fewer waves and did not allow us to examine the long-term effects of chronic disease diagnosis over an extended period. Hence, our dynamic effects study is limited. Fourth, changes in chronic disease conditions affect the persistence of behavioral variation in smoking. Still, because there were many missing values for disease variation in the data, our study did not specifically empirically analyze the effect of disease variation on smoking behavior. Fifth, with the exclusion of females, we were unable to obtain information on the impact of chronic disease diagnoses on female smokers. Finally, this study has limited generalizability to other countries. The above deficiencies warrant further research.

## CONCLUSIONS

This study is one of the few that have examined changes in smoking behavior among LMIC residents, after chronic disease diagnoses. Severe and acute chronic disease diagnosis was found to lower smoking prevalence in the Chinese middle-aged and elderly population over the long-term. The effect of mild and manageable diagnoses on smoking behavior was weaker and not sustainable. These findings contribute to the development of tobacco and chronic disease control policies development in LMICs. Future studies need to explore the effect of chronic disease diagnoses on smoking behavior in different LMICs and further refine the disease type, long-term effects, and mechanisms.

## Data Availability

The data are publicly available from the China Health and Retirement Longitudinal Study (CHARLS) (https://charls.charlsdata.com/).
