# Supplementary material for: The impact of chronic disease diagnoses on smoking behavior change and maintenance: Evidence from China
Source: Tob Induc Dis. 2024 Jan 23;22:10.18332/tid/176947. doi: 10.18332/tid/176947 (PMC10804861; doi:10.18332/tid/176947)
Supplement: Supplementary file 1 [file TID-22-23-s1.pdf]

Supplementary Table 1. The sample size of treatment and control groups across four CHARLS waves (2011, 2013, 2015, 2018).

	2011	2013	2015	2018	Total
**Panel A (Major chronic diseases)**					
Control group sample size	1,141	1,123	1,104	985	4,353
Treatment group sample size	0	18	37	156	211
Total sample size	1,141	1,141	1,141	1,141	4,564
**Panel B (Minor chronic diseases)**					
Control group sample size	1,470	1,391	1,285	985	5,131
Treatment group sample size	0	79	785	485	749
Total sample size	1,470	1,470	1,470	1,470	5,880

Specific sets of underlying two-group two-period difference-in-difference (2×2 DID) estimates in the two-way fixed effects (TWFE) model

In the TWFE framework, the core estimated coefficients are a weighted average of multiple sets of underlying 2×2 DID estimates. They are not actual treatment effects[1]. Specifically, the core estimators in the time-varying difference-in-difference model under TWFE are the weighted average of the 2×2 DID estimates for the four types of subsamples: (i) the 2×2 DID estimates obtained by comparing the pre-treatment group with the untreated group; (ii) the 2×2 DID estimates obtained by comparing the post-treatment group with the untreated group; (iii) the 2×2 DID estimates obtained by comparing the pre-treatment group with the post-treatment group; and (iv) the 2×2 DID estimates obtained by comparing the post-treatment group with the pre-treatment group[2]. Further, the 2×2 DID estimates obtained based on the fourth subsample category lead to lower estimates of TWFE.

1. De Chaisemartin C, D'Haultfoeuille X. Two-Way Fixed Effects Estimators with Heterogeneous Treatment Effects. *Am Econ Rev*. 2020;110(9):2964-2996. doi:10.1257/aer.20181169

2. Goodman-Bacon A. Difference-in-differences with variation in treatment timing. *J Econometrics*. 2021;225(2):254-277. doi:10.1016/j.jeconom.2021.03.014

©2024 Chi X. et al.
